# Behaviors, perceptions, and impact of the COVID-19 pandemic and vaccination on oncology patients in New Mexico with substantial representation of racial minorities and rural residents

**DOI:** 10.1016/j.vaccine.2025.127091

**Published:** 2025-04-11

**Authors:** S. Sasankan, D. Gathers, A. Bellerose, V.S. Pankratz, B. Tawfik

**Affiliations:** a Department of Internal Medicine, Division of Hematology and Oncology, Billings Clinic Cancer Center, 801 N 29th St, Billings, MT 59101, United States; b Department of Internal Medicine, Division of Hematology and Oncology, Duke University Health System, 2301 Erwin Road, Durham, NC 27710, United States; c The Hormel Institute, University of Minnesota, 801 16th Ave NE, Austin, MN 55912, United States; d Department of Internal Medicine, Division of Epidemiology, Biostatistics, and Preventative Medicine, University of New Mexico, 915 Camino de Salud NE, Albuquerque, Albuquerque, NM 87106, United States; e Department of Internal Medicine, Division of Hematology and Oncology, University of New Mexico Comprehensive Cancer Center, 1201 Camino de Salud NE, Albuquerque, NM 87102, United States

**Keywords:** COVID-19, Cancer, Misinformation, Vaccine, Racial minority, Pandemic behaviors

## Abstract

**Introduction:**

The COVID-19 pandemic has affected all sectors of life. This study helps us better understand the perceptions and practices related to the COVID-19 pandemic in the oncology patient population of New Mexico. It also explores patient knowledge regarding safe practices and perceptions regarding vaccination.

**Methods:**

The data for this cross-sectional survey study was collected from July 20, 2021 to September 6, 2021.

**Results:**

There were no significant differences noted in the incidence of COVID-19 infection based on race, comorbidities, or modality of treatment regardless of vaccination status. During the first peak of COVID-19 (Nov 2020 - Jan 2021), most participants followed strict safety precautions (54.2 %), fewer maintained these practices in the months prior to data collection (32.5 %) (Jul 2021 – Aug 2021). Among the participants who had declined vaccination against COVID-19, there were no significant differences based on race, comorbidities or treatment. Those oncology patients receiving treatments at the infusion center were much less vaccine hesitant (8.3 %) compared to those who were not (18.3 %). The odds of COVID-19 infection among patients that were vaccinated was 0.27 times lower than that of unvaccinated patients (95 % CI, 0.12 to 0.63; p 0.001). Vaccine-hesitant respondents reported long-term safety data (*n* = 11, 24.4 %) and physician recommendations (*n* = 10, 22.2 %) were likely to change their minds, but the most common response was that “nothing” would change their mind (*n* = 16, 35.6 %).

**Conclusion:**

The pandemic is a dynamic landscape and physicians need to keep up to date with current guidelines and continue to have conversations with patients regarding strict safety precautions. Having an open discussion with patients regarding vaccine recommendations may help with decreasing vaccine hesitancy.

## 1. Introduction

The respiratory tract infection caused by the severe acute respiratory syndrome coronavirus named SARS-CoV-2 initially emerged in late 2019 [1]. This infection, Coronavirus disease 2019 (COVID-19), was later declared a global pandemic by the World Health Organization in March of 2020 [2]. As of March 2022, two years after the declaration of a global pandemic, there have been 5.95 million deaths worldwide due to this pandemic and 950,000 of these lives have been claimed in the United States alone, the highest number in any country [3]. Patients with cancer are particularly vulnerable to infectious diseases, as antineoplastic treatment compromise the immune system. They usually have other co-morbidities and are of advanced age, leading to increased potential for complications [4]. Recently published literature has also described increased mortality due to COVID-19 among cancer patients [5–7].

Treatment for COVID-19 primarily involves supportive care measures. Preventive care has taken precedence, considering the variability in morbidity and risks for mortality associated with this disease. Starting late 2020 through October 2022, four vaccines were given FDA Emergency Use Authorization. Based on clinical trials, the mRNA vaccines have an efficacy of 95 % after two doses [8,9] and the adjuvanted recombinant spike protein nanoparticle vaccine has an efficacy of 92.6 % [10]^.^

Despite clinical data supporting preventive measures and advising safe practices, the response to the pandemic has included a large amount of misinformation about the virus, safety practices, and the vaccines [11]. The sources of misinformation vary from social media and political figures to retracted scientific articles [12]. Lower socio-demographic status and limited healthcare access have been associated with increased belief in conspiracy theories about COVID-19 and hesitancy regarding vaccination [13]. Minority, rural and economically disadvantaged patients are underrepresented in trials [14,15]. This suggests that studies are needed in these diverse socio-demographic populations to assess cancer patients’ views on COVID-19 and the vaccines.

The University of New Mexico Comprehensive Cancer Center (UNMCCC) is the largest public cancer center in the state of New Mexico. Nearly a quarter (22.6 %) of those residing in New Mexico live in a rural setting; nearly half of the population are of Hispanic ethnicity (49 %), the highest in the country; and nearly one in five of the state’s population live in poverty (18.2 %), the second highest nationally [16,17]. Barriers in healthcare access and poorer cancer outcomes have been documented among these underserved populations [18–21]. This study aimed to investigate the impact of COVID-19 among racial and ethnic minority groups and those living in rural areas served by UNMCCC. It also aimed to further understand the safety practices these patients have followed, perceptions regarding vaccines and the severity of COVID-19 infections.

## 2. Methods

### 2.1. Study design and participants

The study concept was presented and approved at Clinical working group and PRMC committee. It received IRB approval from UNM Health Sciences Office of Research in March of 2021 under exempt research category. This cross-sectional study was conducted in the infusion suite of UNMCCC from July 20, 2021, to September 6, 2021. All cancer patients receiving intravenous infusions at the institution were invited to complete a study survey. Registration staff at the infusion suite distributed paper surveys to patients at the time of check in. Patients anonymously deposited completed surveys in a designated secure box at the registration desk. Because surveys were distributed over a multi-month period, and were subsequently collected anonymously, completed surveys were reviewed by study investigators for duplicates. Duplicates were excluded by comparison of age, zip code, gender, and comorbidity status. Surveys that reflected duplicative responses across these questions were identified as potential duplicates and only the first was retained for primary analyses.

### 2.2. Study instrument

The survey collected basic demographic information which included age, sex, rural residence status, race and ethnicity, and co-morbidities. The co-morbidities of primary interest were diabetes, obesity, chronic lung/heart/kidney disease, all of which have been included in underlying medical conditions associated with higher risk for severe COVID-19 as per CDC. A rural place of residence area was defined as an area with a population less than 50,000 [22]. The severity of symptoms with COVID-19 infections was graded from 0 (asymptomatic) to 4 (critical) as per NIH treatment guidelines [23]. Cancer-related information, including type of cancer, stage, treatment type and any delays experienced in treatment due to the pandemic, was also collected. The safety practices that patients followed, including social isolation and masking, during the time of the peak number of cases, and at the time of survey administration were assessed. Patients were asked to choose between safety practices ranging from strict precautions including rarely leaving the house and contact with nobody outside of household members; moderate precautions comprising masking while out in public and avoiding large gatherings; to normal practices described as continuing with regular life same as prior to the pandemic. Lastly, we collected data regarding the sources of COVID-19 information that were utilized by the study participants, COVID-19 vaccination status, intention to be vaccinated, reasons for not undergoing vaccination, and what would change patients’ perceptions about the vaccine. The final questionnaire (Appendix-1) was formatted in English and Spanish.

### 2.3. Data analysis

Categorical variables were summarized with counts and percentages. Quantitative variables were summarized with means and standard deviations, with medians, 25th and 75th percentiles when appropriate. The primary analyses focused on summarizing the counts and percentages of responses given to the categorical questions posed in the brief survey. Estimates of proportions were obtained from all the surveys, and from subgroups defined by demographic and other participant characteristics. In addition to these simple summaries, potential relationships between factors of interest were explored by performing Fisher’s exact tests. Single-variable logistic regression models were fit to the data to compare the odds of COVID-19 infections between those who did versus those who did not vaccinate, and among groups defined by their levels of preventative precautions. The magnitudes of these associations were described using odds ratio and their corresponding 95 % confidence intervals. A test of symmetry [24] was performed to determine whether participant-reported patterns of COVID-19 preventative precautions changed from the months of peak transmission to the summer months prior to the survey. A single subset analysis was performed within those who were infected with the COVID-19 virus, where a Wilcoxon two-sample test was used to compare the severity of symptoms between those who were versus those who were not vaccinated. *P*-values less than 0.05 were considered statistically significant.

## 3. Results

A total of 376 surveys were collected from July 20, 2021 to September 6, 2021. The mean age of the study participants was 62.9 years, 56 % were female, 36 % Hispanic, 6 % Native American, 69 % of the patients lived in rural zip codes, and 82 % of them were currently undergoing treatment (Table 1). Breast, lung, and colorectal cancer combined constituted 35 % of the respondents. Over the last ten days of the survey period, the numbers of patients who were invited to participate were recorded, along with the number of those who provided a survey response. Of the 265 who were invited, 185 completed a survey, for an estimated response rate of 69.8 %.

There were no significant differences noted in the proportions of respondents who reported symptomatic COVID-19 infections based on demographic data including race and ethnicity, presence of comorbidities, or modality of treatment (Table 2). Only 39 survey respondents (10.3 % of the total) declined COVID-19 vaccination or were hesitant to receive it. A lower proportion of patients receiving IV infusions reported being hesitant 23 out of 276 (8.3 %) than the proportion of those who were not 11 out of 60 (18.3 %, *p* = 0.03), (Table 3).

**Fig. 1.**
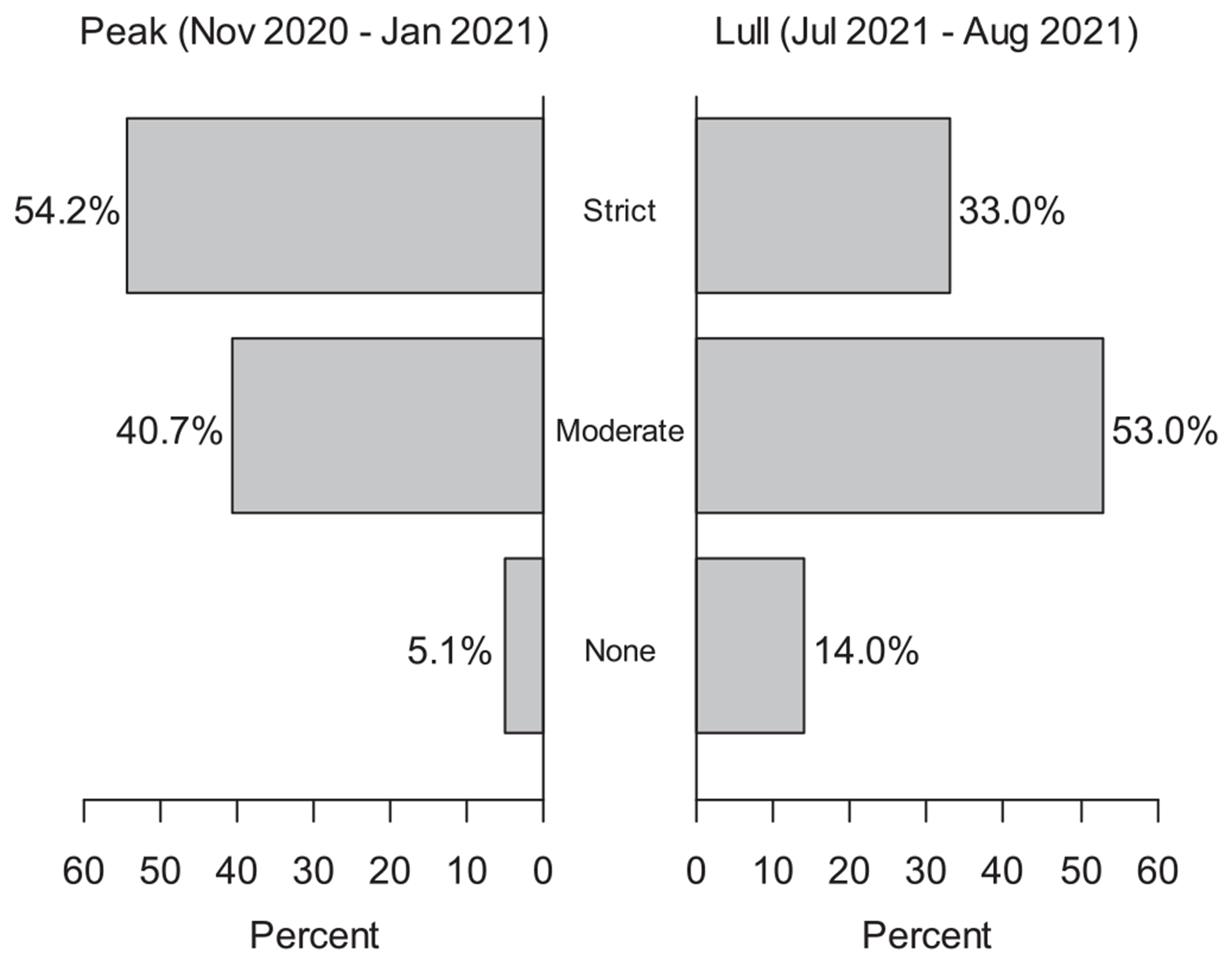
Safety Measures Practiced During the COVID-19 Pandemic at Different Time Points.

While 54.2 % of the respondents followed strict safety measures during the months of peak incidence of COVID-19 (November 2020 – January 2021), this number decreased to 32.5 % in the summer of 2021 (Fig. 1). And 14 % of the respondents had returned to regular life with no specific safety precautions in the month prior to data collection (June 2021 – August 2021). There was a significant change in the reported use of COVID-19 preventative precautions across these two time periods (*p* < 0.001).

Table 4 outlines the distributions of COVID-19 safety precautions taken by survey respondents. At time of peak transmission, more women (58.3 %) than men (48.4 %) took strict precautions (*p* = 0.012). Those who expressed vaccine hesitancy were more likely to take no precautions (23.1 % vs. 3.2 %), and less likely to take strict precautions (43.6 % vs 54.3 %) than those who did not feel hesitant about vaccination (*p* < 0.001). In the month prior to the survey, there were significant differences in the precautions taken among racial/ethnic groups (*p* = 0.014). 28.7 % of Caucasian respondents, 36.2 % of Hispanic respondents, and 42.9 % of other races/ethnicities continued to take strict precautions. Although there was a significant change in use of COVID-19 preventative precautions (p < 0.001, Fig. 1), there was no significant difference in the proportions of individuals reporting COVID-19 infections based on their use of different precautions, either at the time of peak transmission (*p* = 0.18) or in the month prior to survey completion (*p* = 0.68) (Table 4). The odds of COVID-19 infection were nearly equal between those adopting moderate versus strict preventative precautions (OR = 1.02, 95 % CI: 0.48 to 2.17), and were non-significantly higher for those not adopting any precautions versus those adopting strict measures (OR = 2.98, 95 % CI: 0.88 to 10.06) during the time of peak COVID-19 transmission in 2020. The odds of reporting a COVID-19 infection were slightly but not significantly lower among those adopting moderate (OR = 0.72, 95 % CI: 0.34 to 1.57), or no precautions (OR = 0.88, 95 % CI: 0.30 to 2.62) versus those adopting strict measures during the summer months of 2020.

**Fig. 2.**
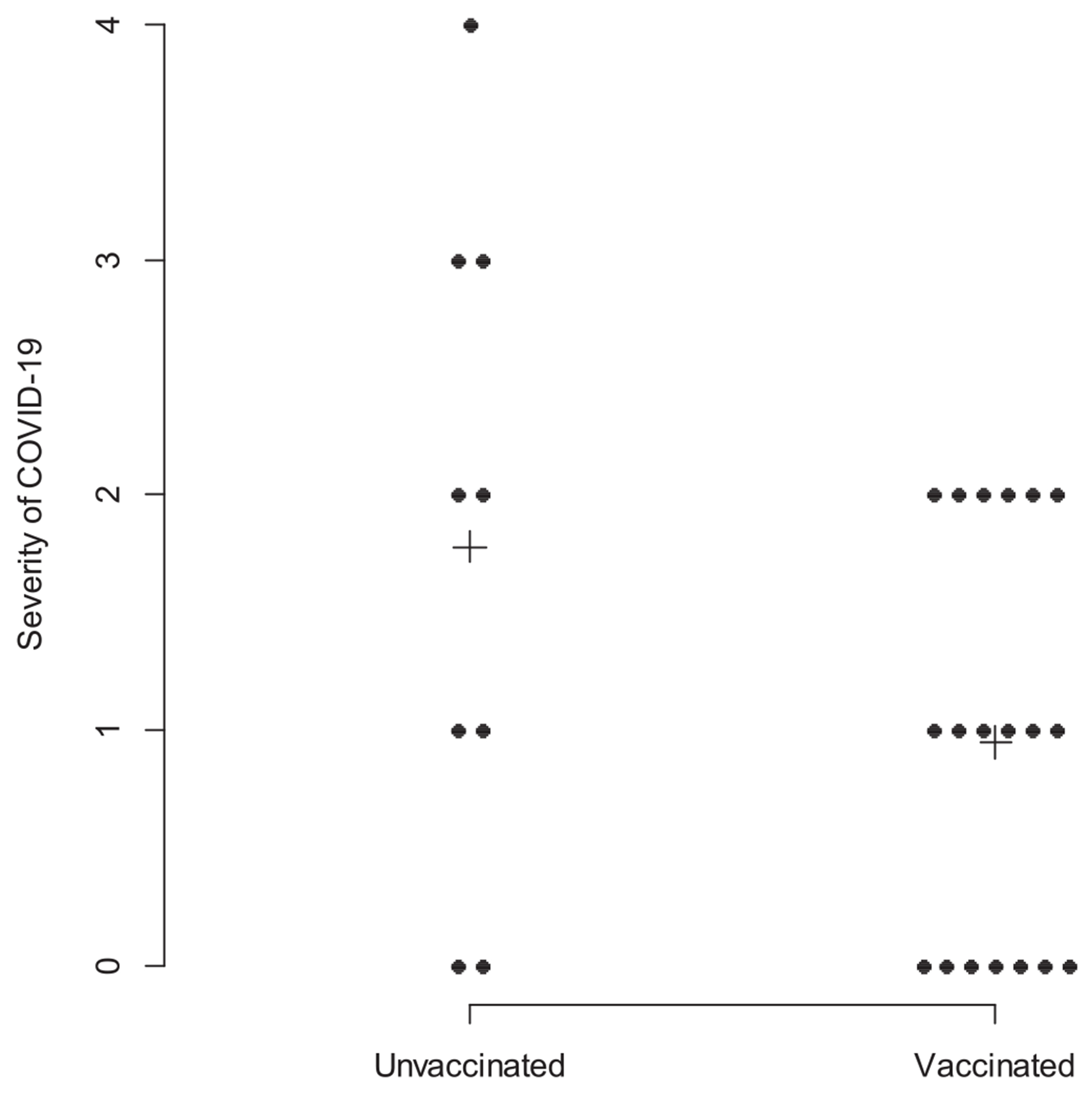
Severity of COVID-19 Infections Based on Vaccination Status. Graded 0–4: 0 – Asymptomatic, 1 – Mild (no shortness of breath), 2 – Moderate (shortness of breath but no supplementary oxygen need), 3 – Severe (Need for supplementary oxygen/hospital stay), 4 – Critical (ICU stay). Mean severity scores, marked by “+”, were 1.78 and 0.95 for unvaccinated and vaccinated individuals, respectively.

The odds of COVID-19 infection among patients that were vaccinated was 0.27 times lower than that of unvaccinated patients (95 % CI: 0.12 to 0.63; *p* = 0.002). Unvaccinated patients had a median severity of COVID-19 symptoms grade of 2, with a 25th percentile grade of 1, and a 75th percentile grade of 3 (mean ± standard deviation = 1.8 ± 1.4, Fig. 2). Vaccinated patients had a median severity of COVID-19 symptoms grade of 1, with a 25th percentile grade of 0 and a 75th percentile grade of 2 (mean ± standard deviation = 0.9 ± 0.8). This difference was not statistically significant via a two-sample Wilcoxon test (*p*-value = 0.12)

Most of the respondents (87.4 %) had already received their vaccines at the time of their response to the survey (Table 5). Of the 294 respondents who provided feedback on sources they listened to for information about COVID-19, 168 (57.1 %) stated that they paid attention to the media, 113 (38.4 %) their physician, 70 (23.8 %) the scientific literature, and 187 (63.6 %) the government, respectively. A significantly lower proportion of respondents who listened to government sources reported being hesitant to receive a COVID-19 vaccine (5.9 % vs. 18.7 %, *p* = 0.001). Similarly, a significantly higher proportion of respondents who listened to government sources had already received a COVID-19 vaccine (92.0 % vs. 79.4 %, *p* = 0.003).

At the time of data collection in July 2021, the vaccines had already received FDA approval under EUA for 6 months. We investigated reasons that might change perceptions about being vaccinated. A total of 33 vaccine-hesitant survey respondents provided responses about what might change their mind about the vaccines. Of these, 7 (21.2 %) reported that long-term safety data and 5 (15.2 %) reported that physician recommendations were likely to change their minds. In this subset of respondents, the most common response was that “nothing” would change their mind (*n* = 15, 45.5 %).

## 4. Discussion

The COVID-19 pandemic has upended daily life globally with significant mortality. Rural patients and those belonging to racial and ethnic minority groups undergoing cancer treatment are at higher risk from COVID-19 and are under-represented in the literature. This study sample contained significant racial/ethnic minority groups (44 %) and rural (64 %) patients, allowing for a better understanding of this group. There were no significant differences noted in the incidence of symptomatic COVID-19 infection based on demographic data. Participants who identified as Hispanics (16 %) were more numerically more hesitant about the vaccine when compared to those who identified as non-Hispanic white (7.7 %) ethnicity, but this was not statistically significant. Those receiving IV infusions were much less hesitant (8.3 %) compared to those who were not (18.3 %), and this may be due to those receiving IV infusions being more immunocompromised and willing to receive the vaccine. This study has demonstrated that while during the first peak of COVID-19, most of the participants followed strict safety precautions (54.2 %), fewer maintained these practices in the months prior to data collection (32.5 %) and this data was collected prior to the federal vaccination mandates. There was a statistically significant gradient in the use of strict precautions by race and ethnicity with 28.7 % of Caucasian respondents, 36.2 % of Hispanic respondents, and 42.9 of other races/ethnicities.

Most of the respondents were already vaccinated at the time (87.4 %) and the incidence of infection was lower among the vaccinated sample 7.3 % (*n* = 22) as compared to unvaccinated sample 22.2 % (*n* = 10). Of those unvaccinated, 35.6 % said “nothing” would change their minds about receiving a vaccine but physician recommendation (22.2 %), vaccine availability (17.8 %), safety data (24.4 %), and scientific data access (8.9 %) could change the minds of the remaining patients. The majority of the participants reported that they rely on media (57.1 %) and the government (63.6 %) as their primary sources of information regarding COVID-19.

The influence of media and government increases their potential to encourage improved public health in general, and specifically through increasing vaccination rate during this pandemic. This has been described domestically, internationally and historically [ [25,26]]. Additionally, the responsibility of the health care team should not be taken lightly in the setting of misinformation. An open discussion about vaccinations between patients and physicians should be encouraged to reinforce data with vaccine recommendation, if appropriate, as these conversations could increase vaccinations among a vulnerable subset of patients. This vulnerable subset not only includes oncology patients but also patients in racial minorities and patients who live in rural areas. Our study focuses on this subset that is often overlooked in scientific literature. It is imperative that physicians and providers keep up-to-date on scientific literature and regulatory updates during a pandemic to be able to provide patients with pertinent evidence-based information.

More than 600,000 people die every year in the US due to cancer, second only to heart disease [27]. COVID was the 3rd leading cause of death among all adults at the time of this study in 2021 [28]. Delays in cancer diagnosis and treatment can lead to considerable morbidity and mortality, but only 6 % of the study sample reported treatment delays. This is remarkable as literature described interruptions in treatment of up to 77.5 %, primarily due to provider or system related variables [29]. The pandemic presented a rapidly changing landscape and safety precautions followed by patients change based on number of cases in community. This has been described by increased use of masks and reduced use of public transit during the peak incidence in November–December 2020, that was followed by relaxation of precautions as case numbers decreased [30].

It is important to note the limitations of this study. This was a cross-sectional study at one Comprehensive Cancer Center and may not be applicable to other institutions. It was conducted after the first two waves of COVID-19 and six months after the vaccines were available to the public, so it may not be applicable to other time points. It is possible, patients were vaccinated or infected with later surges, which were not captured. In the dynamic landscape related to the pandemic, this study represents only a snapshot. The information collected, including safety practices, comorbidities, and primary cancer diagnosis, was limited by recall bias. Furthermore, participation was voluntary, and it is possible that patients who elected not to participate were hesitant to declare their beliefs or may have only spoken languages other than English or Spanish. Some patients receiving infusions may have had benign conditions and thus were not immunocompromised and may not have felt a significant need to take strict precautions against COVID-19. While the population served by the cancer center has a high poverty rate, this was not directly measured during this study. Small differences between groups were likely missed due to the limited size of this study sample.

## 5. Conclusion

This study describes the safety practices and vaccine perceptions in New Mexico, which has a substantial representation of minority racial or ethnic groups and rural patients, with cancer during the COVID-19 pandemic. It is important for physicians to discuss current scientific data, recommend vaccination, and encourage an open conversation with patients regarding vaccination to better support them and reduce mortality and morbidity. Future research could look at tailored vaccine educational interventions in patients with cancer.

## Supplementary Material

Appendix

## Figures and Tables

**Table 1:** Demographic and clinical characteristics of the 376 individuals with cancer being treated in the Infusion suite of the University of New Mexico Comprehensive Cancer Center.**

Age (16 missing responses)	Mean	Standard Deviation

Years	62.9	12.1
Gender Identified (7 missing responses)	N	%
Male	163	44 %
Female	206	56 %
Race and Ethnicity (8 missing responses)		
Non Hispanic White/Caucasian	186	51 %
Hispanic/Latino	132	36 %
Native American	21	6 %
African American	7	2 %
Asian	2	1 %
Other/Multiple	20	5 %
Zip Code (16 missing responses)		
Rural	247	69 %
Urban	113	31 %
Comorbidities (11 missing responses)		
Absent	250	68 %
Present	115	32 %
Treatment (28 missing responses)		
IV Infusion or Injection	286	82 %
Transfusions/Hydration	37	11 %
Radiation Therapy	19	5 %
Oral Medications	71	20 %
Cancer Type* (13 missing responses)		
Bladder	13	4 %
Breast	53	15 %
Colorectal	39	11 %
Esophagus	5	1 %
Kidney	8	2 %
Leukemia/MDS	26	7 %
Liver	20	6 %
Lung	38	10 %
Melanoma	7	2 %
Pancreatic	25	7 %
Prostate	28	8 %
Stomach	7	2 %
Uterine/ovarian/Peritoneal	25	7 %
Multiple myeloma/Lymphoma	50	14 %
Neuroendocrine Tumor	9	2 %
Sarcoma	5	1 %
Brain	3	1 %
Blood Disorders	6	2 %
Other Type**	16	4 %

* Some patients listed more than one type of cancer or condition

** Other types of cancer included Anal, Appendiceal, Gallbladder, Pituitary, Squamous Cell (2), Testicular (2), Head and Neck (2), Thyroid (2), Skin, Spinal Tumor, Lipomatosis, Carcinoma unspecified

**Table 2:** COVID-19 infections reported by patients with cancer, according to key demographic characteristics.^2^

	N^1^	Number (%) of Infections	p-value^2^
Gender Identified (20 missing responses)			
Male	154	14 (9.1)	0.86
Female	202	20 (9.9)	
Ethnicity (19 missing responses)			
Non Hispanic White	183	14 (7.7)	0.43
Hispanic	126	15 (11.9)	
Other	48	5 (10.4)	
Zip Code (28 missing responses)			
Rural	239	24 (10.0)	1.00
Urban	109	10 (9.2)	
Comorbidities (23 missing responses)			
Absent	244	20 (8.2)	0.32
Present	109	13 (11.9)	
Treatment (39 missing responses)			
IV Infusion or Injection: No	60	3 (5.0)	0.23
Yes	277	30 (10.8)	
Transfusions/Hydration: No	302	31 (10.3)	0.55
Yes	35	2 (5.7)	
Radiation Therapy: No	319	32 (10.0)	1.00
Yes	18	1 (5.6)	
Oral Medications: No	270	24 (8.9)	0.26
Yes	67	9 (13.4)	

1 Number of participants providing responses for both demographic and COVID-19 infection questions.

2 Fisher’s exact test.

**Table 3:** COVID-19 vaccine hesitancy by patients with cancer, according to key demographic characteristics.^1^, ^2^

	N^1^	Number (%) Reporting No current interest inCOVID-19 Vaccination	p-value^2^
	Gender Identified (23 missing responses)		
Male	153	16 (10.5)	1.00
Female	200	21 (10.5)	
	Ethnicity (21 missing responses)		
Non Hispanic White	182	14 (7.7)	0.07
Hispanic	125	20 (16.0)	
Other	48	5 (10.4)	
	Zip Code (30 missing responses)		
Rural	237	21 (8.9)	0.13
Urban	109	16 (14.7)	
	Comorbidities (25 missing responses)		
Absent	237	30 (12.7)	0.21
Present	114	9 (7.9)	
Treatment (40 missing responses)			
IV Infusion or	60	11 (18.33)	0.03
Injection: No	276	23 (8.3)	
Yes			
Transfusions/Hydration: No	301	29 (9.6)	0.38
Yes	35	5 (14.3)	
Radiation Therapy:	318	33 (10.4)	1.00
No	18	1 (5.6)	
Yes			
Oral Medications: No	266	26 (9.8)	0.66
Yes	70	8 (11.4)	

1 Number of participants providing responses for both demographic and COVID-19 infection questions.

2 Fisher’s exact test.

**Table 4:** Use of COVID-19 safety precautions taken by patients with cancer, according to key demographic characteristics: At peak COVID-19 transmission, and in the month prior to survey.^1^, ^2^

	Peak COVID-19 Transmission (Nov 2020-Jan 2021) N^1^ (%)				Lull COVID-19 Transmission (Jul 2021-Aug 2021) N^1^ (%)			

	No Precautions	Moderate Precautions	Strict Precautions	p-value^2^	No Precautions	Moderate Precautions	Strict Precautions	p-value^2^
Gender Identified		11 missing responses				16 missing responses		
Male	14 (8.8)	68 (42.8)	77 (48.4)	0.012	26 (16.8)	83 (53.6)	46 (29.7)	0.37
Female	5 (2.4)	81 (39.3)	120 (58.3)		25 (12.2)	109 (53.2)	71 (34.6)	
sienceEthnicity		11 missing responses				15 missing responses		
Non Hispanic White	13 (7.0)	77 (41.4)	96 (51.6)	0.15	37 (20.0)	95 (51.4)	53 (28.7)	0.014
Hispanic	4 (3.1)	57 (44.2)	68 (52.7)		10 (7.9)	71 (55.9)	46 (36.2)	
Other	2 (4.0)	14 (28.0)	34 (68.0)		4 (8.2)	24 (49.0)	21 (42.9)	
Zip Code		19 missing responses				24 missing responses		
Rural	11 (4.5)	101 (41.2)	133 (54.3)	0.52	34 (14.1)	130 (53.9)	77 (32.0)	0.90
Urban	8 (7.1)	47 (42.0)	57 (50.9)		17 (15.3)	57 (51.4)	37 (33.3)	
Comorbidities		14 missing responses				18 missing responses		
Absent	13 (5.3)	112 (45.3)	122 (49.4)	0.046	39 (15.9)	123 (50.2)	83 (33.9)	0.18
Present	5 (4.4)	37 (32.2)	73 (63.5)		11 (9.7)	67 (59.3)	35 (31.0)	
Treatment		28 missing responses				33 missing responses		
IV Infusion or Injection:	4 (6.5)	27 (43.6)	31 (50.0)	0.69	13 (21.0)	27 (43.6)	22 (35.5)	0.16
No	15 (5.2)	115 (40.2)	156 (54.6)		36 (12.8)	154 (54.8)	91 (32.4)	
Yes								
Transfusions/Hydration:	17 (5.5)	129 (41.5)	165 (53.1)	0.71	45 (14.7)	164 (53.4)	98 (31.9)	0.49
No	2 (5.4)	13 (35.1)	22 (59.5)		4 (11.1)	17 (47.2)	15 (41.7)	
Yes								
Radiation Therapy: No	18 (5.5)	135 (41.0)	176 (53.5)	0.93	47 (14.5)	169 (52.0)	109 (33.5)	0.53
Yes	1 (5.3)	7 (36.8)	11 (57.9)		2 (11.1)	12 (66.7)	4 (22.2)	
Oral Medications: No	14 (5.1)	113 (40.8)	150 (54.2)	0.76	38 (14.0)	144 (52.9)	90 (33.1)	0.95
Yes	5 (7.0)	29 (40.9)	37 (52.1)		11 (15.5)	37 (52.1)	23 (32.4)	
Vaccine Hesitancy		20 missing responses				23 missing responses		
Received/Interested	10 (3.2)	135 (42.6)	172 (54.3)	<0.001	41 (13.1)	172 (54.8)	101 (32.2)	0.10
Declined/Hesitant	9 (23.1)	13 (33.3)	17 (43.6)		10 (25.6)	17 (43.6)	12 (30.8)	
COVID-19 Infection		18 missing responses				19 missing responses		
No	14 (4.3)	133 (41.1)	177 (54.6)	0.18	45 (13.9)	175 (54.2)	103 (31.9)	0.68
Yes	4 (11.8)	13 (38.2)	17 (50.0)		5 (14.7)	16 (47.1)	13 (38.2)	

1 Number of participants providing responses for both demographic and COVID-19 infection questions.

2 Fisher’s exact test.

**Table 5:** View of COVID-19 vaccines, and vaccination status, by sources of information relied on by study respondents.^1^

	COVID-19 Vaccination Status N^1^ (%)			View of COVID-19 Vaccines N^1^ (%)		

Source of Information	Not Vaccinated	Vaccinated	p-value^2^	Hesitant	Not Hesitant	p-value^2^
Media						
No	18 (14.3)	108 (85.7)	0.48	14 (11.1)	112 (88.9)	0.85
Yes	19 (11.3)	149 (88.7)		17 (10.1)	151 (89.9)	
Physician						
No	24 (13.3)	157 (86.7)	0.72	20 (11.1)	161 (89.0)	0.85
Yes	13 (11.5)	100 (88.5)		11 (9.7)	102 (90.3)	
Scientific articles						
No	26 (11.6)	198 (88.4)	0.41	20 (8.9)	204 (91.1)	0.12
Yes	11 (15.7)	59 (84.3)		11 (15.7)	59 (84.3)	
Government						
No	22 (20.6)	85 (79.4)	0.003	20 (18.7)	87 (81.3)	0.001
Yes	15 (8.0)	172 (92.0)		11 (5.9)	176 (94.1)	

1 Number of participants providing responses for both demographic and COVID-19 infection questions (there were a total of 82 individuals who did not provide information about the combinations of these factors).

2 Fisher’s exact test.

## Data Availability

Data will be made available on request.

## Appendix A. Supplementary data

Supplementary data to this article can be found online at https://doi.org/10.1016/j.vaccine.2025.127091.
