# Supplementary material for: Behaviors, perceptions, and impact of the COVID-19 pandemic and vaccination on oncology patients in New Mexico with substantial representation of racial minorities and rural residents
Source: Vaccine. Author manuscript; Available in PMC 2026 Mar 2. (PMC12951894; doi:10.1016/j.vaccine.2025.127091)
Supplement: Appendix [file NIHMS2146247-supplement-Appendix.docx]

**APPENDIX-1**

**QUESTIONNAIRE – PLEASE SUBMIT ONLY ONE COMPLETED FORM PER PATIENT**

1. Gender:
   1. Male
   2. Female

1. Age: ___________

1. Race/Ethnicity:
   1. Caucasian
   2. Hispanic/Latinx
   3. African American
   4. Native American
   5. Asian
   6. Other (Specify):

1. Zip code of current primary address: ______________

1. Do you have any serious health conditions other than cancer (Diabetes/Obesity/Lung, Heart or Chronic kidney problems):
   1. Yes
   2. No

1. Type of Cancer:
   1. Bladder
   2. Breast
   3. Colorectal
   4. Esophagus
   5. Kidney
   6. Leukemia
   7. Liver
   8. Lung
   9. Melanoma
   10. Lymphoma
   11. Pancreatic/Gall bladder
   12. Prostate
   13. Stomach
   14. Uterine/Ovarian
   15. Multiple Myeloma
   16. Other (Specify):

1. What cancer treatment are you currently undergoing? (Choose multiple options, if applicable)
   1. IV infusions or Injections (Chemotherapy/ Immunotherapy/Targeted therapy/Hormonal therapy)
   2. Transfusions/ Hydration
   3. Radiation
   4. Oral pills (Chemotherapy/ Targeted therapy/Hormonal therapy)

1. Safety measures during peak COVID:
   1. Strictly staying at home and rarely leaving the house, meeting nobody outside of household members
   2. Masking while out, meeting small groups of people but avoiding large gatherings
   3. Continuing with regular life same as before COVID-19 pandemic

1. Safety measures in the past month:
   1. Strictly staying at home and rarely leaving the house, meeting nobody outside of household members
   2. Masking while out, meeting small groups of people but avoiding large gatherings
   3. Continuing with regular life same as before COVID-19 pandemic

1. Have you had COVID-19 infection with at least one positive test (If no, proceed to question 13):
   1. Yes
   2. No

1. Symptoms associated with confirmed COVID-19 infection
   1. Asymptomatic
   2. Mild (no shortness of breath)
   3. Moderate (shortness of breath but no supplementary oxygen need)
   4. Severe (Need for supplementary oxygen/hospital stay)
   5. Critical (ICU stay)

1. Received Antibody infusion (Bamlavinimab) treatment for COVID infection:
   1. Yes
   2. No

1. Interruption or delay in cancer treatment due to pandemic or COVID Illness:
   1. Yes
   2. No

1. COVID vaccine
   1. Received it
   2. Registered for it/Interested
   3. Hesitant
   4. Not interested

1. If you have not received or registered for the vaccine, when do you plan on registering for it
   1. 1-3 months
   2. 3-6 months
   3. 6-12 months
   4. 1-2 years
   5. Never

1. If hesitant or NOT interested in receiving the vaccine, what are your concerns? (Choose multiple options, if applicable)
   1. Lack of long-term safety data
   2. I do not believe the vaccine works
   3. I think COVID is overblown or not real
   4. Worried about side effects
   5. I already had COVID and don’t require the vaccine
   6. Other (specify):

1. If hesitant or NOT interested in receiving the vaccine, what would change your mind about it? (Choose multiple options, if applicable)
   1. Your oncologist/PCP recommending it
   2. The Cancer center having the vaccine available
   3. Long term safety data
   4. Access to current scientific data and a discussion about that data
   5. Nothing
   6. Other (specify):

1. COVID related information source: (choose multiple options, if applicable)
   1. Media/social media
   2. Oncologist/Physician
   3. Scientific paper/Health Care Organization video
   4. NM Department of Health or CDC

1. Have you received the Flu vaccine in the past two years
   1. Yes
   2. No
